# Thanks to Reviewers 2014

**DOI:** 10.5334/pb.ba

**Published:** 2014-12-31

**Authors:** Editorial Team

**Affiliations:** 1 Psychologica Belgica, Belgium

## Abstract

All manuscripts published in *Psychologica Belgica* have been assessed conscientiously and unselfishly by expert reviewers. The quality of our journal totally depends on their valuable and constructive criticisms to the authors. Both the editors and the authors highly appreciate the input and dedication of all our reviewers. Many thanks.

**Baars Martine**, Hogeschool Rotterdam, the Netherlands

**Baldwin Carryl**, George Mason University, USA

**Boen Filip**, KULeuven, Belgium

**Brebels Lieven**, KULeuven, Campus Brussels, Belgium

**Brenning Katrijn**, Ghent University, Belgium

**Content Alain**, Free University of Brussels (ULB), Belgium

**De Mol Jan**, Université Catholique de Louvain, Belgium

**De Neys Wim**, Université Paris Descartes, France

**Dardenne Benoit**, University of Liège, Belgium

**Dehon Hedwige**, University of Liège, Belgium

**Delrue Jochen**, Ghent University, Belgium

**Demeyer Ineke**, Ghent University, Belgium

**Fagnant Annick**, University of Liège, Belgium

**Feys Marjolein**, Ghent University, Belgium

**Gaudreau Patrick**, University of Ottawa, Canada 

**Goossens Lien**, Ghent University, Belgium

**Hansenne Michel**, University of Liège, Belgium

**Hansez Isabelle**, University of Liège, Belgium

**Hellin Joost**, AZ Nikolaas, Sint-Niklaas, Belgium

**Jelicic Marko**, Maastricht University, The Netherlands

**Kalyuga Slava**, University of New South Wales (UNSW), Australia

**Klimstra Theo**, Univeristy of Tilburg, the Netherlands

**Lahaye Magali**, Université Catholique de Louvain, Belgium

**Lens Willy**, KULeuven, Belgium

**Leunissen Joost**, University of Southampton, UK

**Magis David**, University of Liège, Belgium

**Mikolajczak Moira**, Université catholique de Louvain, Belgium

**Monseur Christian**, University of Liège, Belgium

**Mueller Astrid**, Medizinische Hochschule Hannover, Germany

**Nader-Grosbois Nathalie**, Université Catholique de Louvain, Belgium

**Nelemans Stefanie**, Universiteit Utrecht, the Netherlands

**Panizza Daniele**, Georg August Universität, Göttingen, Germany

**Quoidbach Jordi**, University Pompeu Fabra, Barcelona, Spain

**Reinders Folmer Chris**, Erasmus University Rotterdam, the Netherlands

**Schaefer Alexandre**, Durham University, UK 

**Spronk Marjolein**, KULeuven, Belgium 

**Stiévenart Marie**, Université Catholique de Louvain, Belgium

**Tan E.S.**, University of Amsterdam, The Netherlands

**Theuns Peter**, Free University of Brussels (VUB), Belgium

**van Alphen Bas**, Free University of Brussels (VUB), Belgium

**Van den Bergh Bram**, Erasmus University Rotterdam, the Netherlands

**Vanheule Stijn**, Ghent University, Belgium

**Van Overwalle Frank**, Free University of Brussels (VUB), Belgium

**van Tiel Bob**, Radboud University Nijmegen, the Netherlands

**Vantilborgh Tim**, Free University of Brussels (VUB), Belgium

**Verhaeghen Paul**, Georgia Institute of Technology, USA

**Verschuere Bruno**, University of Amsterdam, The Netherlands

**Willems Kim**, Free University of Brussels (VUB), Belgium

**Winterstein Gregoire**, Université Paris Diderot, France

**Wood Alex**, Stirling Management School, UK

